# Risk Factors on the Incidence and Prognostic Effects of Colorectal Cancer With Brain Metastasis: A SEER-Based Study

**DOI:** 10.3389/fonc.2022.758681

**Published:** 2022-03-18

**Authors:** Quan Chen, Le He, Yuhong Li, Chenghai Zuo, Mengxia Li, Xiaofeng Wu, Chengxiu Pu, Xiang Xu, Rongrui Tang, Yanli Xiong, Juan Li

**Affiliations:** ^1^ Cancer Center, Daping Hospital, Army Medical University, Chongqing, China; ^2^ Department of Stem Cell & Regenerative Medicine, State Key Laboratory of Trauma, Burn and Combined Injury, Daping Hospital, Army Medical University, Chongqing, China; ^3^ Department of Gastroenterology, Chongqing General Hospital, University of Chinese Academy of Sciences, Chongqing, China; ^4^ Department of Neurosurgery and Key Laboratory of Neurotrauma, Southwest Hospital, the Third Military Medical University, Chongqing, China; ^5^ Department of Neurosurgery, University-Town Hospital of Chongqing Medical University, Chongqing, China

**Keywords:** colorectal cancer, brain metastasis, incidence, risk factor, prognosis, SEER

## Abstract

**Background:**

Colorectal cancer (CRC) with brain metastases (BM) is uncommon and often diagnosed at a late stage. The aims of this study were to identify the clinical factors that can influence the incidence of CRC patients with BM (CRCBM) and to investigate the impact of clinical factors and therapies on the outcomes of CRCBM.

**Methods:**

Between 2010 and 2018, patients with CRCBM were enrolled under the Surveillance, Epidemiology, and End Results (SEER) program. Multivariable logistic and Cox regression models were used to identify risk factors and prognostic factors of BM. Kaplan–Meier curve and log-rank test were used to evaluate overall survival (OS) and tumor-specific survival (CSS) of CRCBM patients.

**Results:**

A total of 195 (0.34%) CRC patients initially diagnosed with BM were included for analysis. The positive level of CEA, pN2a-b, and additional organ metastases were positively associated with developing BM from the CRC cohort (*p* < 0.05). The median OS and CSS of the BM patients were both 4.0 months, while the corresponding survival time in CRC patients without BM was 14.0 and 16.0 months, respectively (HR = 2.621, 95% CI = 2.061–3.333 for CSS; HR = 2.556, 95% CI = 2.026–3.225 for OS; log rank *p* < 0.001, each). Only systematic treatment was independently associated with better survival (*p* < 0.05, each).

**Conclusions:**

Although the overall prognosis of CRCBM patients was extremely poor, the positive level of CEA, pN2a-b, and distant metastases could be bad risk factors for the incidence of CRCBM. In addition, only systematic treatment was found to be a negative prognostic factor for CRCBM patients. These related factors can provide more valuable reference for clinical individualized treatments.

## Introduction

Colorectal cancer (CRC) is the third most common cancer with an incidence rate of 38.7 per 100,000, and the mortality rate was 13.9 per 100,000 persons based on the most recent record (1). Distant metastases occur frequently in CRC patients; however, brain metastasis (BM) with an incidence of 1%–4% in metastatic CRC (mCRC) is relatively rarer than other common carcinomas such as lung, liver, breast or kidney cancers (2–4). Unfortunately, some recent studies have found that the incidence of BM in CRC (CRCBM) has been increasing in recent decades (5). Indeed, some neural treatments can achieve prolonged intracranial tumor control in selected patients (6). However, brain or cerebral imaging is not routinely recommended in clinical guidelines for mCRC at risk, which means that BM is widely diagnosed only when symptomatic, hence often at a late stage. Mackenzie reported that the rate of BM from CRC with brain-related symptoms is 76%, and more than a half of them suffered from severe neurologic complications (7). Therefore, CRCBM cannot be regarded as a rare clinical problem with less attention; continuous efforts are thus warranted for the prediction of CRCBM.

According to previous studies, some researchers have worked on identifying risk factors for the prediction of CRCBM, which suggested that some risk factors including primary location of rectal cancer, lung metastasis, performance status, number of extracranial metastases, tumor stage, grade, and some gene mutations were associated with the occurrence of BM (2, 8–10). However, the clinical application of these conclusions is limited by a small sample size and lack of wide validation.

Compared to other solid malignancies, patients with CRCBM had remarkably poor prognosis, with a median survival of only about 5.0 months upon diagnosis (11–13). The treatment of mCRC has dramatically improved in recent years with additional radical surgery and targeted therapies, while effective therapies for CRCBM patients remain largely based on primary tumors, which include surgery, radiosurgery, whole brain radiotherapy, and systemic therapy (6, 14, 15). Moreover, some clinicopathological and molecular signatures were reported to be positively associated with longer survival in patients with CRCBM, but the retrospective nature of these studies limits the conclusions (8, 16, 17).

In this study, we collected data from CRCBM patients through a nationwide database and used the clinicopathological data aiming to determine the predictive risk factors for occurrence and the clinical factors and therapeutic effects on prognosis of CRCBM patients.

## Materials and Methods

### Data Source and Eligibility Criteria

The patients’ records were collected from the Surveillance, Epidemiology, and End Results (SEER) database, which was founded by the National Cancer Institute (NCI), providing clinicopathological data including incidence, therapy, and survival data of many tumors. The dataset contains more than 11,865,152 entries, including 839,695 patients of brain metastatic neoplasm with initial primary cancer up to 2018. The analysis data were downloaded from the SEER database containing information on cancer patients diagnosed from 1975 to 2018, released on April 2021, based on the November 2020 submission [Incidence - SEER Research Plus Data, 9 Registries, Nov 2020 Sub (1975–2018)].

**Figure 1:**
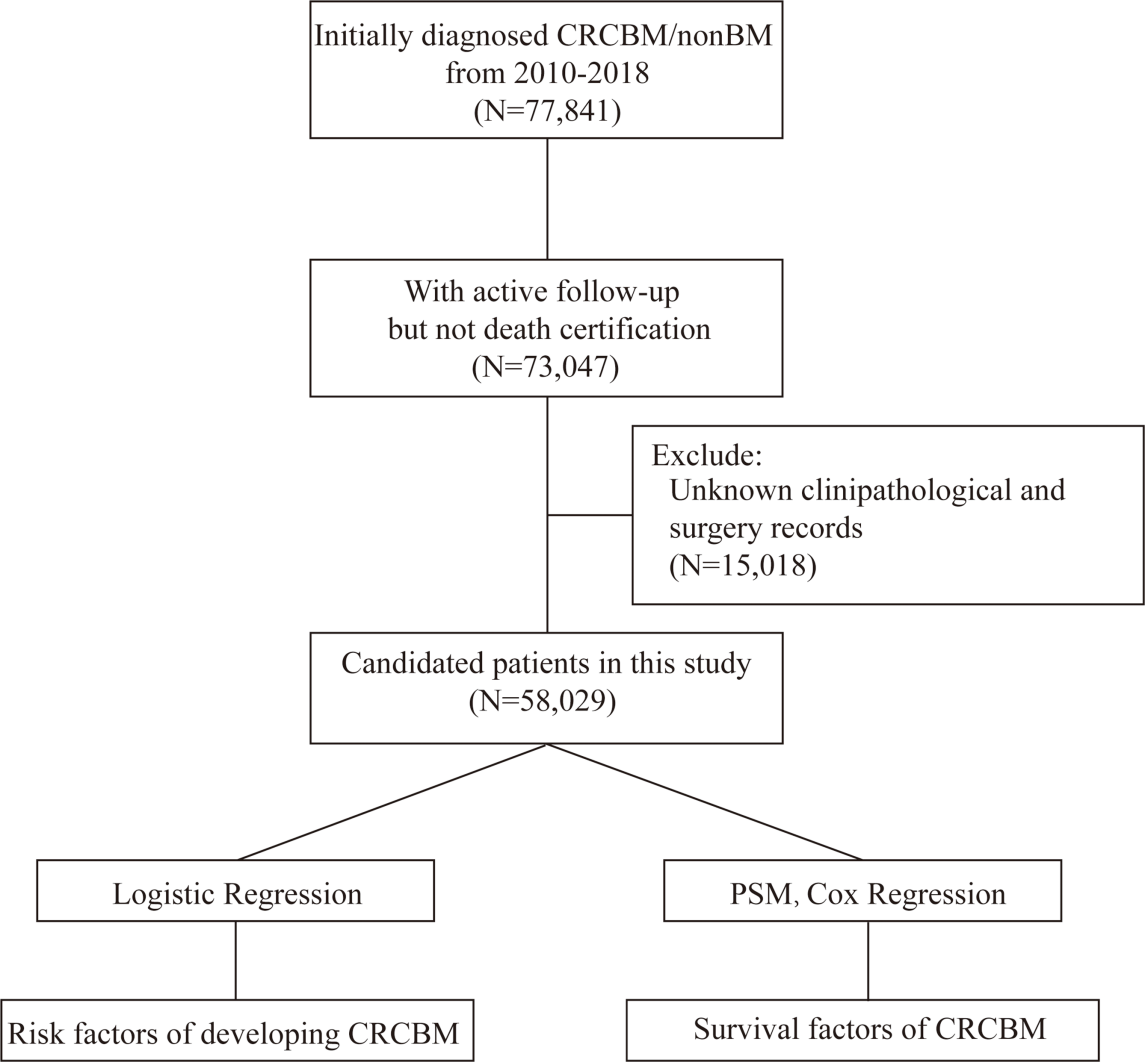
Flowchart for selection and analysis procedure in the study. Initially, we derived CRC patients with exact records of BM from the SEER database, and then removed the patients that did not meet the criteria. Finally, univariable and multivariable logistic and Cox regression analysis were performed to obtain the individual variables that affect the incidence and prognosis of CRCBM, respectively.

Patients who were diagnosed with CRC from 2010 to 2018 were screened out from the database. Patients whom we selected met the following criteria: (I) patients who were initially diagnosed as CRC with no other sequent records of primary malignant cancers; (II) patients with unknown records of BM; (III) CRC patients with histological ICD-O-3 codes, including 8000–8152, 8154–8231, 8243–8245, 8250–8576, 8940–8950, and 8980–8981 (18) and (IV) The patients who were recorded death within 30 days after a confirmed diagnosis. The flowchart of this study is presented in **Figure 1**.

### Study Variables

The selected patients were divided into one group with BM and another group without BM. Clinical baseline variables including age, gender, race, location, grade, histology, pT, pN, bone metastases, liver metastases, lung metastases, CEA, perineural invasion, surgery, radiotherapy, and systematic therapy were used in the current study. The primary location on patients was further classified into three groups: right-sided colon (C18.0–18.4), left-sided colon (C18.5–18.7), and rectum (C19.9 or C20.9). The histology variable was classified as “Adenocarcinoma (AC, ICD-O-3: 8140 to 8147, 8210 to 8211, 8220 to 8221, 8260 to 8263)”, “Mucinous adenocarcinoma (MC, ICD-O-3: 8480, 8481)”, “Signet ring cell carcinoma (SRCC, ICD-O-3: 8490)”, and “Other”. Grade was defined by the following codes: well differentiated (Grade I), moderately differentiated (Grade II), poorly differentiated (Grade III), undifferentiated (Grade IV), and unknown grade. The pathological stage was determined referring to the 8th edition of the American Joint Committee on Cancer tumor-node-metastasis (TNM) staging system (19). In order to test the influence on patients with additional distant metastasis organs, we stratified the patients with BM by the number of additional distant metastasis organs according to the SEER Met records. We removed the cases who received systematic therapy both before and after surgery and surgery both before and after systematic therapy due to the small scale of numbers. Patients with unknown records of surgery were removed, and unknown records of chemotherapy, radiotherapy, and systematic treatments were defined as “None/Unknown”, as per SEER definition. Both OS and CSS were used to analyze the survival outcomes.

### Statistical Analysis

Descriptive statistics were used to summarize demographics, and a chi-square test was performed to compare categorical variables between cases with and without BM as baseline clinical characteristics. Multivariable logistic regression analysis was used to assess the risk factors of BM from CRC.

The patients in this paper were adjusted using propensity score matching (PSM) in order to minimize the differences among study covariates, which could become confounding factors to evaluate the effect of risk factors and treatments in a nonrandomized level. We initially performed logistic regression to find out the significant influential factors and then identified a new set of unmatched patients according to these significant covariates (20, 21). By classifying the nearest neighbor propensity score into two groups (CRC patients with BM and those without BM) within a defined limit of caliper, we yielded two well-matched patient sets. After PSM, we compared the survival difference between patients with CRCBM and those with no BM.

According to the SEER cause-specific death classification, patients in this study who died due to this cancer were censored at the time of death for time-to-cancer analyses. Meanwhile, patients who died from any cause were also censored at the time of death for time-to-overall analyses. The cancer-specific survival (CSS) and overall survival (OS) were analyzed by Kaplan–Meier survival curves and compared by the log-rank test. Prognosis and the therapeutic effect were analyzed using Cox proportional hazards regression models.

The SEER Stat (National Cancer Institute, Bethesda, MD, USA; version 8.3.9) was used to download data in this study. PSM was performed by R MatchIt. Statistical analyses were performed using IBM SPSS Statistics version 22 (IBM Corporation, Armonk, NY, USA) and R software (version 4.0.3). *p* < 0.05 was considered statistically significant. The procedure in the study is shown in **Figure 1**.

## Results

### Characteristics of Study Patients

In this study, we have obtained 58,029 cases with initial primary CRC who were diagnosed from 2010 to 2018, of which 195 cases (0.34%) were diagnosed with BM. The clinical characteristics of CRC and the chi-square test for comparison among CRC patients with BM and those without BM are presented in **Table 1**. There were significant differences in grade, histology, pT, pN, bone metastases, liver metastases, lung metastases, CEA, surgery, and radiotherapy (*p* < 0.001, each). In pathological characteristics, CRC patients with BM were found to be with unknown grade (37.43% vs. 10.99%), pT (43.59% vs. 6.56%), and pN (36.92% vs. 4.95%), and much more often accompanied by metastases of bone (21.03% vs. 0.92%), liver (61.03% vs. 13.71%), and lung (51.79% vs. 4.56%). Meanwhile, there was a significantly higher positive level of CEA in patients with BM (60.00% vs. 28.37%). Compared with patients without BM in the difference in treatment, CRCBM patients were found to receive less surgery (28.72% vs. 86.78%) but more radiotherapy (66.15% vs. 13.31%). However, the variables age, race, gender, location, perineural invasion, and systematic therapy did not have a significant difference (**Table 1**, *p* > 0.05).

**Table 1:** Demographic and clinical characteristics of the patients in the cohort.

	BM (%)	Non-BM (%)	*p* ^*^
Age (years)			0.127
<50	31 (15.90%)	7,602 (13.14%)	
50–59	54 (27.69%)	13,076 (22.61%)	
60–69	51 (26.16%)	14,704 (25.42%)	
70–79	33 (16.92%)	11,981 (20.72%)	
≥80	26 (13.33%)	10,471 (18.11%)	
Race			0.252
White	156 (80.00%)	43,692 (75.55%)	
Black	22 (11.28%)	6,955 (12.02%)	
Other^†^	17 (8.72%)	7,187 (12.43%)	
Gender			0.160
Male	91 (46.67%)	29,902 (51.70%)	
Female	104 (53.33%)	27,932 (48.30%)	
Location			0.057
Right side	83 (42.56%)	25,340 (43.82%)	
Left side	46 (23.59%)	16,942 (29.29%)	
Rectum	66 (33.85%)	15,552 (26.89%)	
Grade			<0.001
Grade I	2 (1.03%)	4,479 (7.75%)	
Grade II	76 (38.97%)	37,371 (64.62%)	
Grade III	38 (19.49%)	8,093 (13.99%)	
Grade IV	6 (3.08%)	1,534 (2.65%)	
Unknown	73 (37.43%)	6,357 (10.99%)	
Histology			<0.001
AC	172 (88.21%)	51,579 (89.18%)	
MC	4 (2.05%)	3,833 (6.63%)	
SRCC	4 (2.05%)	615 (1.07%)	
Other	15 (7.69%)	1,807 (3.12%)	
pT			<0.001
T1–2	34 (17.44%)	17,519 (30.29%)	
T3–4	76 (38.97%)	36,520 (63.15%)	
Unknown	85 (43.59%)	3,795 (6.56%)	
pN			<0.001
N0–N1b	95 (48.72%)	46,957 (81.19%)	
N2a–2b	28 (14.36%)	8,014 (13.86%)	
Unknown	72 (36.92%)	2,863 (4.95%)	
Bone metastases			<0.001
No/Unknown	154 (78.97%)	57,302 (99.08%)	
Yes	41 (21.03%)	532 (0.92%)	
Liver metastases			<0.001
No/Unknown	76 (38.97%)	49,905 (86.29%)	
Yes	119 (61.03%)	7,929 (13.71%)	
Lung metastases			<0.001
No/Unknown	94 (48.21%)	55,196 (95.44%)	
Yes	101 (51.79%)	2,638 (4.56%)	
CEA			<0.001
Negative/Unknown	78 (40.00%)	41,425 (71.63%)	
Positive	117 (60.00%)	16,409 (28.37%)	
Perineural Invasion			0.174
None/Unknown	182 (93.33%)	52,322 (90.47%)	
Present	13 (6.67%)	5,512 (9.53%)	
Surgery			<0.001
None/unknown	139 (71.28%)	7,648 (13.22%)	
Performed	56 (28.72%)	50,186 (86.78%)	
Radiotherapy			<0.001
None/unknown	66 (33.85%)	50,133 (86.68%)	
Performed	129 (66.15%)	7,701 (13.32%)	
Systematic therapy			0.096
None	127 (65.13%)	36,560 (63.22%)	
Before surgery	7 (3.60%)	4,449 (7.69%)	
After surgery	61 (31.27%)	16,825 (29.09%)	

AC, adenocarcinoma; MC, Mucinous adenocarcinoma; SRCC, Signet ring cell carcinoma.

^∗^p-values were made by chi-square test.

^†^Other = American Indian/AK Native, and Asian/Pacific Islander.

### The Risk Factors for Developing BM in CRC

We performed univariable and multivariable logistic regressions to predict the occurrence of CRCBM by clinical and pathological variables. Univariable logistic regression showed that CRC patients with moderately and poorly differentiated grades (Grades II and III) and undifferentiated grade (Grade IV), pN2a-b, other histology, higher positive level of CEA, bone metastases, liver metastases, and lung metastases were positively associated with BM (**Supplementary Table 1**, *p* < 0.05, each). Based on the results of multivariable logistic regression analysis, pN2a-b (*p* = 0.048), higher positive level of CEA (*p* < 0.001), bone metastases (*p* < 0.001), liver metastases (*p* = 0.005), and lung metastases (*p* < 0.001) were associated with significantly higher risk for BM from CRC, while the diagnosed age, race, gender, pT, and perineural invasion were found not to be significantly correlated with CRCBM (**Table 2**, *p* > 0.05).

**Table 2:** Multivariable logistic regression for analyzing the risk factors for brain metastasis from CRC before PSM.

	OR	95% CI	*p* ^∗^
Age (years)			0.495
<50	1		
50–59	1.330	0.621–2.850	
60–69	0.927	0.421–2.042	
70–79	0.671	0.273–1.650	
≥80	1.100	0.459–2.637	
Race			0.170
White	1		
Black	0.857	0.440–1.671	
Other^†^	0.377	0.135–1.051	
Gender			0.598
Male	1		
Female	1.138	0.705–1.837	
Location			0.135
Right side	1		
Left side	1.085	0.630–1.867	
Rectum	0.553	0.280–1.094	
Grade			0.076
Grade I	1		
Grade II	1.546	0.368–6.495	
Grade III	3.425	0.779–15.059	
Grade IV	2.624	0.422–16.331	
Unknown	2.321	0.494–10.895	
Histology			0.293
AC	1		
MC	0.202	0.028–1.475	
SRCC	1.607	0.367–7.024	
Other	0.405	0.054–3.018	
pT			0.238
T1–2	1		
T3–4	0.690	0.373–1.278	
Unknown	–	–	
pN			0.048
N0–N1b	1		
N2a–N2b	1.760	1.006–3.077	
Unknown	–	–	
Bone metastasis			<0.001
No/Unknown	1		
Yes	4.855	2.213–10.654	
Liver metastasis			0.005
No/Unknown	1		
Yes	2.412	1.304–4.462	
Lung metastasis			<0.001
No/Unknown	1		
Yes	8.786	4.868–15.857	
CEA			<0.001
Negative/Unknown	1		
Positive	3.037	1.746–5.284	
Perineural Invasion			0.519
Not present/Unknown	1		
Present	0.803	0.412–1.566	

AC, adenocarcinoma; MC, Mucinous adenocarcinoma; SRCC, Signet ring cell carcinoma.

^∗^p-values were calculated by multivariable logistic regression after adjusted by age, gender, race, location, grade, histology, pT, pN, bone metastases, liver metastases, lung metastases, CEA, and perineural invasion.

^†^Other = American Indian/AK Native, and Asian/Pacific Islander.

### Survival Comparison Between CRC Patients With BM and Those Without BM

According to the results of univariable logistic regression (**Supplementary Table 1**, *p* < 0.05), we identified nine factors that could affect the outcomes of treatment, namely, grade, histology, pN, bone metastases, liver metastases, lung metastases, CEA, surgery, and radiotherapy, and used these factors to generate a propensity score. After PSM, we obtained 167 patients with BM and 321 patients with no BM in this study, and none of the above factors between the two groups was found to be significantly different (**Supplementary Table 2**, *p* > 0.05).

**Figure 2:**
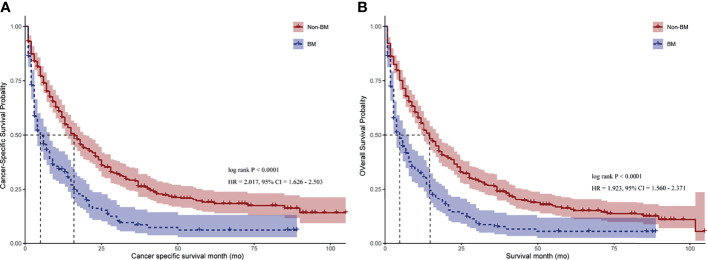
Kaplan–Meier curves for CRC patients with BM and those with no BM. **(A)** Cancer-specific survival (CSS) for CRC patients with BM and those with no BM. **(B)** Overall survival (OS) for CRC patients with BM and those with no BM. HR = hazard ratio. 95% CI = 95% confidence interval. *p*-value was calculated by log-rank test.

Furthermore, we compared the survival difference among patients after using PSM. The median CSS and OS of CRC patients without BM were significantly higher than that of patients with BM (16.0 months vs. 4.0 months for CSS, **Figure 2A**, *p* < 0.001; 14.0 months vs. 4.0 months for OS, **Figure 2B**, *p* < 0.001). Consistently, CRC patients with BM have worse CSS than those without BM after univariate Cox model (HR = 2.017; 95% CI = 1.626–2.503; *p* < 0.001, **Supplementary Table 3**). Meanwhile, after modeling all variables in multivariable Cox analysis, CRC patients with BM have worse CSS when compared with those without BM (HR = 2.621; 95% CI = 2.061–3.333; *p* < 0.001; **Table 3**). Similar results were also obtained when analyzed by OS (HR = 2.556; 95% CI = 2.026–3.225; *p* < 0.001; **Table 3**).

**Table 3:** Multivariable analyses using Cox models associated with CSS and OS for patients with CRC after PSM.

	CSS			OS		
	HR	95% CI	*p* ^∗^	HR	95% CI	*p* ^∗^
Brain metastasis			<0.001			<0.001
None	1			1		
Yes	2.621	2.061–3.333		2.556	2.026–3.225	
Age(years)			<0.001			<0.001
<50	1			1		
50–59	1.449	1.020–2.058		1.464	1.038–2.065	
60–69	1.462	1.035–2.066		1.470	1.049–2.059	
70–79	1.803	1.224–2.656		1.963	1.352–2.850	
≥80	3.933	2.588–5.975		4.231	2.827–6.332	
Race			0.123			0.028
White	1			1		
Black	1.314	0.959–1.799		1.479	1.101–1.986	
Other^†^	1.310	0.901–1.905		1.225	0.843–1.782	
Gender			0.345			0.121
Male	1			1		
Female	0.900	0.724–1.120		0.846	0.685–1.045	
Location			0.31			0.039
Right side	1			1		
Left side	0.680	0.495–0.934		0.752	0.557–1.016	
Rectum	0.755	0.570–1.000		0.723	0.550–0.951	
Grade			0.006			0.023
Grade I	1			1		
Grade II	0.554	0.249–1.233		0.506	0.239–1.070	
Grade III	0.962	0.425–2.179		0.797	0.370–1.718	
Grade IV	0.628	0.227–1.742		0.480	0.180–1.278	
Unknown	0.569	0.255–1.268		0.538	0.254–1.142	
Histology			0.107			0.403
AC	1			1		
MC	0.893	0.511–1.558		0.865	0.509–1.468	
SRCC	0.862	0.352–2.109		0.772	0.319–1.868	
Other	0.526	0.313–0.882		0.693	0.437–1.098	
pT			0.143			0.144
T1–2	1			1		
T3–4	0.939	0.651–1.354		1.022	0.716–1.458	
Unknown	1.229	0.885–1.709		1.288	0.933–1.778	
pN			0.149			0.114
N0–N1b	1			1		
N2a–N2b	1.446	0.968–2.160		1.470	1.002–2.157	
Unknown	1.141	0.891–1.460		1.130	0.890–1.436	
Bone metastasis			0.013			0.018
No/Unknown	1			1		
Yes	1.459	1.083–1.965		1.419	1.062–1.898	
Liver metastasis			<0.001			<0.001
No/Unknown	1			1		
Yes	2.054	1.570–2.687		1.872	1.446–2.422	
Lung metastasis			0.033			0.076
No/Unknown	1			1		
Yes	1.297	1.021–1.647		1.233	0.979–1.553	
CEA			0.316			0.261
Negative/Unknown	1			1		
Positive	0.883	0.693–1.126		0.874	0.692–1.105	
Perineural Invasion			0.846			0.865
Not present/Unknown	1			1		
Present	1.050	0.641–1.721		1.042	0.648–1.676	
Surgery			0.023			0.018
None/unknown	1			1		
Performed	0.591	0.376–0.929		0.593	0.384–0.914	
Radiotherapy			0.123			0.224
None/unknown	1			1		
Performed	1.213	0.949–1.552		1.157	0.915–1.463	
Systematic therapy			0.034			0.019
None	1			1		
Before surgery	0.461	0.237–0.896		0.460	0.239–0.887	
After surgery	0.686	0.472–0.998		0.658	0.460–0.940	

AC, adenocarcinoma; MC, Mucinous adenocarcinoma; SRCC, Signet ring cell carcinoma.

^∗^p-values were calculated by multivariable Cox regression after adjusted by brain metastasis, age, gender, race, location, grade, histology, pT, pN, bone metastasis, liver metastasis, lung metastasis, CEA, and perineural invasion.

^†^Other = American Indian/AK Native, and Asian/Pacific Islander.

### Survival Benefits of Clinical Factors and Therapies

**Figure 3:**
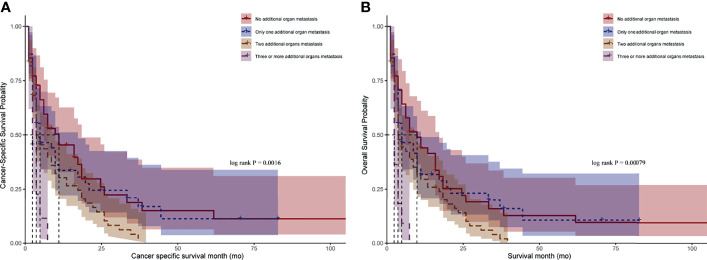
Kaplan–Meier curves for CRC patients with BM after stratified by the number of additional distant metastasis organs. **(A)** Comparison of cancer-specific survival (CSS). **(B)** Comparison of overall survival (OS). *p*-value was calculated by log-rank test.

We stratified the patients with BM by the number of additional distant metastasis organs and found that the median CSS for CRCBM patients with zero, one, two, and three or more additional distant metastasis organs were 9.0 months, 4.0 months, 3.0 months, and 2.0 months, respectively, with statistically significant differences (**Figure 3A**, *p* < 0.05). A similar result can also be found in OS (**Figure 3B**, *p* < 0.05).

**Figure 4:**
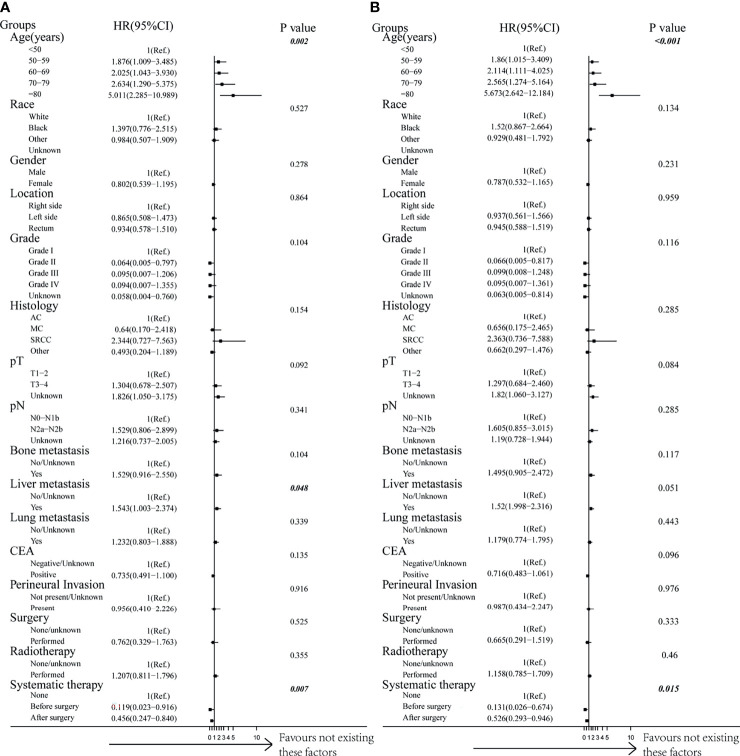
Forest plot of multivariable Cox proportional hazard ratio (HR) and 95% CI for cancer-specific survival and overall survival of patients with BM after PSM. **(A)** Comparison of cancer-specific survival (CSS). **(B)** Comparison of overall survival (OS). *p*-value was calculated by multivariable Cox regression after adjusted by variables including age, gender, race, location, grade, histology, pT, pN, bone metastases, liver metastases, lung metastases, CEA, perineural invasion, surgery, radiotherapy, and systematic therapy.

By performing both univariable and multivariable Cox regression analyses, we predicted the survival benefits of clinical factors and effective therapies for CRC patients with BM. Interestingly, the results demonstrated that a younger age of diagnosis and having received systematic therapies could mean a longer CSS and OS (**Figures 4A, B**, **Table 4** and **Supplementary Table 4**, *p* < 0.05, each). However, clinicopathological factors, the presence of additional distant metastasis organs, and receiving surgery or radiotherapy do not have an influence on both CSS and OS in CRCBM patients (**Figures 4A, B**, **Table 4** and **Supplementary Table 4**, *p* > 0.05, each).

**Table 4:** Multivariable Cox analysis of prognostic factors associated with CSS and OS for CRCBM patients after PSM.

	CSS			OS		
	HR	95% CI	*p* ^*^	HR	95% CI	*p* ^∗^
Age (years)			0.002			<0.001
<50	1			1		
50–59	1.876	1.009–3.485		1.860	1.015–3.409	
60–69	2.025	1.043–3.930		2.114	1.111–4.025	
70–79	2.634	1.290–5.375		2.565	1.274–5.164	
≥80	5.011	2.285–10.989		5.673	2.642–12.184	
Race			0.527			0.134
White	1			1		
Black	1.397	0.776–2.515		1.520	0.867–2.664	
Other^†^	0.984	0.507–1.909		0.929	0.481–1.792	
Gender			0.278			0.231
Male	1			1		
Female	0.802	0.539–1.195		0.787	0.532–1.165	
Location			0.864			0.959
Right side	1			1		
Left side	0.865	0.508–1.473		0.937	0.561–1.566	
Rectum	0.934	0.578–1.510		0.945	0.588–1.519	
Grade			0.104			0.116
Grade I	1			1		
Grade II	0.064	0.005–0.797		0.066	0.005–0.817	
Grade III	0.095	0.007–1.206		0.099	0.008–1.248	
Grade IV	0.094	0.007–1.355		0.095	0.007–1.361	
Unknown	0.058	0.004–0.760		0.063	0.005–0.814	
Histology			0.154			0.285
AC	1			1		
MC	0.640	0.170–2.418		0.656	0.175–2.465	
SRCC	2.344	0.727–7.563		2.363	0.736–7.588	
Other	0.493	0.204–1.189		0.662	0.297–1.476	
pT			0.092			0.084
T1–2	1			1		
T3–4	1.304	0.678–2.507		1.297	0.684–2.460	
Unknown	1.826	1.050–3.175		1.820	1.060–3.127	
pN			0.341			0.285
N0–N1b	1			1		
N2a–N2b	1.529	0.806–2.899		1.605	0.855–3.015	
Unknown	1.216	0.737–2.005		1.190	0.728–1.944	
Bone metastasis			0.104			0.117
No/Unknown	1			1		
Yes	1.529	0.916–2.550		1.495	0.905–2.472	
Liver metastasis			0.048			0.051
No/Unknown	1			1		
Yes	1.543	1.003–2.374		1.520	1.998–2.316	
Lung metastasis			0.339			0.443
No/Unknown	1			1		
Yes	1.232	0.803–1.888		1.179	0.774–1.795	
CEA			0.135			0.096
Negative/Unknown	1			1		
Positive	0.735	0.491–1.100		0.716	0.483–1.061	
Perineural Invasion			0.916			0.976
Not present/Unknown	1			1		
Present	0.956	0.410–2.226		0.987	0.434–2.247	
Surgery			0.525			0.333
None/unknown	1			1		
Performed	0.762	0.329–1.763		0.665	0.291–1.519	
Radiotherapy			0.355			0.460
None/unknown	1			1		
Performed	1.207	0.811–1.796		1.158	0.785–1.709	
Systematic therapy			0.007			0.015
None	1			1		
Before surgery	0.119	0.023–0.916		0.131	0.026–0.674	
After surgery	0.456	0.247–0.840		0.526	0.293–0.946	

AC, adenocarcinoma; MC, Mucinous adenocarcinoma; SRCC, Signet ring cell carcinoma.

^∗^p-values were calculated by multivariable Cox regression after adjusted by brain metastasis, age, gender, race, location, grade, histology, pT, pN, bone metastasis, liver metastasis, lung metastasis, CEA, and perineural invasion.

^†^Other = American Indian/AK Native, and Asian/Pacific Islander.

## Discussion

As promoted in 2020, headways in the treatment of metastatic diseases, especially from liver and lung metastases, have increased the survival rate of CRC patients in recent decades (1, 22, 23). However, little is known about CRCBM due to its low incidence rate. Unfortunately, the prevalence of BM in patients with CRC is significantly increasing (24); however, magnetic resonance imaging (MRI) or computed tomography (CT) is not a routine brain examination for CRC patients during follow-up. Consequently, missed diagnosis of CRCBM may occur, and the reported incidence rate of CRCBM may be lower than the actual incidence rate (25). In addition, some studies with small samples reported a wide variation in incidence. These differences in incidence may be due to bias among the enrolled patients in different studies. Therefore, there is an urgent need to use the latest sufficiently large patient cohort to reevaluate rare BM in these CRC patients. In this nationwide population-based study, CRC patients with BM were identified in the SEER database and PSM was conducted to adjust for confounders for the first time. Considering that SEER is a large database, strict quality control is implemented and data are updated annually to ensure data accuracy (26), and our results proved to be very reliable.

Compared to other primary tumor sources such as lung cancer and breast cancer, which are prone to brain metastases, BM in CRC is relatively rare (25). Although precise incidence rates of CRCBM patients had been found in sporadic studies, they were based on a limited number of cases. Consequently, the incidence of CRCBM has been found to range from 0.1% to 4% (10, 27–30). This discrepancy may be due to the differences in the enrolled patients. Based on SEER data, CRCBM patients are relatively rare, with only 0.34% of CRC patients diagnosed with BM, which was consistent with other research cohorts (12, 27). However, the true incidence rate may be higher, as these assessments were often limited to patients with symptoms requiring treatment and because many studies reported only the presence or absence of BM at the time of initial diagnosis; further information on whether the patient developed BM during or after treatment was not provided.

The incidence risk factors for these patients were also reported at the time of initial diagnosis. Compared with non-BM patients, the proportion of BM patients with synchronous bone, liver, and lung metastasis was higher, and the proportion of only single-organ metastasis was also higher in CRCBM patients than in non-CRCBM patients. In addition, the positive level of CEA was also higher in BM patients, and these results suggest that CRCBM patients may suffer from a higher tumor burden. Due to the higher incidence of distant metastases, especially multiple unresectable distant metastases, CRCBM patients received local treatment with low surgical rate and high radiotherapy rate. Several risk factors were finally identified and required attention, including lymph node metastasis at pN2a-b, positive level of CEA, and additional distant metastases (lung, liver, or bone). These are important predictors to help clinicians determine the occurrence of BM when the above characteristics are present in CRC patients. Therefore, patients initially diagnosed with the above risk factors may need to pay more attention to the possibility of brain metastases, and brain examination, *via* MRI or CT, should be considered to avoid missed diagnosis of BM for CRC patients with high risk factors during follow-up (1).

CRCBM patients usually have a very poor prognosis, with a median survival of only about 5.0 months (11, 12). Our study is consistent with other studies in that CRCBM patients also had a significantly shorter median OS and CSS than BM-free CRC patients. We further confirmed that the survival of CRC patients with metastasis to BM only was better than that with synchronous metastasis to other organs. In addition, the number of metastatic organs is also a significant prognostic factor, wherein the greater the number of additional distant metastasis organs, the lower the chance of survival. However, the results demonstrated that a younger age of diagnosis and having received systematic therapies could mean a longer CSS and OS, while other factors, including surgery, radiotherapy, and pathological clinical factors, did not have a significant impact on survival of CRCBM patients. Interestingly, although pN2a-b, positive level of CEA, and several distant metastases can be used as significant predictors to predict the risk of BM, these factors could not be used as prognostic factors. Also, the presence of additional distant metastasis organs, surgery, and radiotherapy do not influence both CSS and OS in CRCBM patients.

Of all the treatments, only systematic treatment actually extended CSS and OS in CRCBM patients in our study. Consistent with our research, the recent studies reported that the interval between first CRC diagnosis and diagnosis of BM is increasing due to constantly improving systematic treatments (14, 31). Even more interesting, CRCBM exhibits elevated mutational signatures of homologous recombination deficiency and mismatch repair deficiency, which means a promising effective systematic treatment of target and immune therapy (32). It also means that more alternative multimodality systematic treatments may reduce the occurrence of BM and prolong the survival of CRCBM patients. According to the time sequence between systemic treatment and surgery, systematic treatment can be stratified into before and after surgery, which both promoted the survival of CRCBM patients in our study. Since most systematic treatments before surgery were considered as neoadjuvant treatments, we can assume that CRCBM patients can benefit from neoadjuvant treatment. In addition, systematic treatments after surgery included systematic treatment or adjuvant treatments for mCRC; hence, further studies are needed to determine which of the two treatments, if not both, could benefit patients with CRCBM.

The study has several limitations. Firstly, there are inevitable inherent biases in any retrospective study. Secondly, the SEER database includes only the US population, so the results of this study may not be appropriate for populations in other countries or regions and should be interpreted with caution. Thirdly, although SEER provides a large platform for depth and longitudinal analysis of cancer patients, it is still considered administrative data with limitations and some records are not detailed enough for clinical application such as information on clinicopathological factors (i.e., TN stage and pathological grade) and the detailed records of treatment methods (i.e., surgery, radiotherapy chemotherapy, systematic treatment methods, and sequence of treatments) for CRCBM patients, which are crucial for analysis. As an important example, it was found that the majority of CRCBM patients who received radiotherapy did not benefit from this treatment, and one of the possible reasons is that the site of radiotherapy (whether it was the primary tumor or the metastatic tumor, or both) was unclear.

In conclusion, the incidence rate of CRCBM patients is 0.34% according to the above analysis. Although there are some limitations, our study reported the positive level of CEA, pN2a-b, and distant metastases as risk factors, while only systematic treatment was reported as a prognostic factor for CRCBM patients based on the SEER database. These factors could be used as a reference in clinical decision-making for individualized treatment.

## Data Availability Statement

The datasets generated and analyzed during the current study are available in the Surveillance, Epidemiology, and End Results (http://www.seer.cancer.gov/). Further inquiries can be directed to the corresponding authors.

## Ethics Statement

Written informed consent was obtained from the individual(s) for the publication of any potentially identifiable images or data included in this article.

## Author Contributions

LH redesigned the paper and revised most of the paper according to the comments of reviewers. QC, RT, YX, and JL conceived and coordinated the study. QC, YL, and CZ wrote and revised the paper. LH, ML, XW, and CP performed and analyzed the data. XX and YX offered technical or material support, critical reading, and text revisions. All authors reviewed the results and approved the final version of the manuscript.

## Funding

This work was supported by the National Key R&D Program (2018YFC1313400) and the National Natural Science Foundation of China (Grant Number: 81902671).

## Conflict of Interest

The authors declare that the research was conducted in the absence of any commercial or financial relationships that could be construed as a potential conflict of interest.

## Publisher’s Note

All claims expressed in this article are solely those of the authors and do not necessarily represent those of their affiliated organizations, or those of the publisher, the editors and the reviewers. Any product that may be evaluated in this article, or claim that may be made by its manufacturer, is not guaranteed or endorsed by the publisher.

## Supplementary Material

Supplementary Table 1 Univariate logistic regression for analyzing the risk factors for brain metastasis from CRC.

Supplementary Table 2 Baseline demographic and clinical characteristics before and after propensity score matching.

Supplementary Table 3 Univariable analyses using Cox models associated with CSS and OS for patients with CRC after PSM.

Supplementary Table 4 Univariable Cox analysis of prognostic factors associated with CSS and OS for CRCBM patients after PSM.

## Abbreviations

AC, adenocarcinoma; BM, brain metastasis; CEA, carcinoembryonic antigen; CI, confidence interval; CRCBM, colorectal cancer with BM; CRC, colorectal cancer; CSS, cancer-specific survival; CT, computed tomography; HR, hazard ratio; mCRC, metastatic colorectal cancer; MC, mucinous adenocarcinoma; MRI, magnetic resonance imaging; OS, overall survival; pM, the absence or presence of pathological distant metastatic lesions; pN, the absence or presence of pathological regional lymph node metastases; pT, the extent of pathological primary tumor; PSM, propensity score matching; SEER, Surveillance, Epidemiology, and End Results; SRCC, signet ring cell carcinoma; TNM, tumor-node-metastasis.
