# Supplementary material for: Risk Factors on the Incidence and Prognostic Effects of Colorectal Cancer With Brain Metastasis: A SEER-Based Study
Source: Front Oncol. 2022 Mar 18;12:758681. doi: 10.3389/fonc.2022.758681 (PMC8971714; doi:10.3389/fonc.2022.758681)
Supplement: Supplementary Table 1 — Univariate logistic regression for analyzing the risk factors for brain metastasis from CRC. [file Table_1.docx]

**Supplementary Table 1** Univariate logistic regression for analyzing the risk factors for brain metastasis from CRC.

|  | OR | 95%CI | P |
| --- | --- | --- | --- |
| Age(years) |  |  | 0.132 |
| <50 | 1 |  |  |
| 50-59 | 1.013 | 0.651-1.576 |  |
| 60-69 | 0.851 | 0.544-1.330 |  |
| 70-79 | 0.675 | 0.544-1.330 |  |
| ≥80 | 0.609 | 0.361-1.026 |  |
| Race |  |  | 0.257 |
| White | 1 |  |  |
| Black | 0.886 | 0.576-1.385 |  |
| Other† | 0.662 | 0.401-1.094 |  |
| Gender |  |  | 0.161 |
| Male | 1 |  |  |
| Female | 1.223 | 0.923-1.622 |  |
| Location |  |  | 0.059 |
| Right side | 1 |  |  |
| Left side | 0.829 | 0.578-1.189 |  |
| Rectum | 1.296 | 0.937-1.791 |  |
| Grade |  |  | <0.001 |
| Grade I | 1 |  |  |
| Grade II | 4.554 | 1.118-18.550 |  |
| Grade III | 10.515 | 2.536-43.607 |  |
| Grade IV | 8.759 | 1.766-43.445 |  |
| Unknown | 25.717 | 6.308-104.840 |  |
| Histology |  |  | <0.001 |
| AC | 1 |  |  |
| MC | 0.313 | 0.116-0.844 |  |
| SRCC | 1.950 | 0.721-5.273 |  |
| Other | 2.489 | 1.466-4.228 |  |
| pT |  |  | 0.735 |
| T1-2 | 1 |  |  |
| T3-4 | 1.072 | 0.715-1.607 |  |
| Unknown | - | - |  |
| pN |  |  | 0.011 |
| N0-N1b | 1 |  |  |
| N2a-N2b | 1.727 | 1.132-2.634 |  |
| Unknown | - | - |  |
| Bone metastasis |  |  | <0.001 |
| No/Unknown | 1 |  |  |
| Yes | 28.676 | 20.110-40.892 |  |
| Liver metastasis |  |  | <0.001 |
| No/Unknown | 1 |  |  |
| Yes | 9.855 | 7.383-13.154 |  |
| Lung metastasis |  |  | <0.001 |
| No/Unknown | 1 |  |  |
| Yes | 22.482 | 16.930-29.853 |  |
| CEA |  |  | <0.001 |
| Negative/Unknown | 1 |  |  |
| Positive | 3.787 | 2.842-5.046 |  |
| Perineural Invasion |  |  | 0.176 |
| Not present/Unknown | 1 |  |  |
| Present | 0.678 | 0.386-1.191 |  |
| Surgery |  |  | <0.001 |
| None/unknown | 1 |  |  |
| Performed | 0.061 | 0.045-0.084 |  |
| Radiotherapy |  |  | <0.001 |
| None/unknown | 1 |  |  |
| Performed | 12.724 | 9.449-17.134 |  |
| Systematic therapy |  |  | 0.108 |
| None | 1 |  |  |
| Before surgery | 0.453 | 0.211-0.970 |  |
| After surgery | 1.044 | 0.769-1.417 |  |

Abbreviations: AC adenocarcinoma, MC Mucinous adenocarcinoma, and SRCC Signet ring cell carcinoma.

† Other=American Indian/AK Native, and Asian/Pacific Islander
