# Supplementary material for: Risk Factors on the Incidence and Prognostic Effects of Colorectal Cancer With Brain Metastasis: A SEER-Based Study
Source: Front Oncol. 2022 Mar 18;12:758681. doi: 10.3389/fonc.2022.758681 (PMC8971714; doi:10.3389/fonc.2022.758681)
Supplement: Supplementary Table 2 — Baseline demographic and clinical characteristics before and after propensity score matching. [file Table_2.docx]

**Supplementary Table 2** Baseline demographic and clinical characteristics before and after propensity score matching.

|  | Before PSM | | | After PSM | | |
| --- | --- | --- | --- | --- | --- | --- |
|  | BM  (%) | Non-BM  (%) | P^*^ | BM  (%) | Non-BM  (%) | P^*^ |
| Age(years) |  |  | 0.127 |  |  | 0.660 |
| <50 | 31(15.90%) | 7602(13.14%) |  | 22(13.17%) | 59(18.38%) |  |
| 50-59 | 54(27.69%) | 13076(22.61%) |  | 43(25.75%) | 74(23.05%) |  |
| 60-69 | 51(26.16%) | 14704(25.42%) |  | 47(28.14%) | 83(25.86%) |  |
| 70-79 | 33(16.92%) | 11981(20.72%) |  | 30(17.96%) | 55(17.13%) |  |
| ≥80 | 26(13.33%) | 10471(18.11%) |  | 25(14.98%) | 50(15.58%) |  |
| Race |  |  | 0.252 |  |  | 0.501 |
| White | 156(80.00%) | 43692(75.55%) |  | 132(79.04%) | 239(74.45%) |  |
| Black | 22(11.28%) | 6955(12.02%) |  | 21(12.58%) | 52(16.20%) |  |
| Other^†^ | 17(8.72%) | 7187(12.43%) |  | 14(8.38%) | 30(9.35%) |  |
| Gender |  |  | 0.160 |  |  | 0.691 |
| Male | 91(46.67%) | 29902(51.70%) |  | 78(46.71%) | 156(48.59%) |  |
| Female | 104(53.33%) | 27932(48.30%) |  | 89(53.29%) | 165(51.41%) |  |
| Location |  |  | 0.057 |  |  | 0.641 |
| Right side | 83(42.56%) | 25340(43.82%) |  | 68(40.71%) | 133(41.43%) |  |
| Left side | 46(23.59%) | 16942(29.29%) |  | 37(22.16%) | 60(18.69%) |  |
| Rectum | 66(33.85%) | 15552(26.89%) |  | 62(37.13%) | 128(39.88%) |  |
| Grade |  |  | <0.001 |  |  | 0.361 |
| Grade I | 2(1.03%) | 4479(7.75%) |  | 1(0.60%) | 10(3.11%) |  |
| Grade II | 76(38.97%) | 37371(64.62%) |  | 66(39.52%) | 129(40.19%) |  |
| Grade III | 38(19.49%) | 8093(13.99%) |  | 35(20.96%) | 53(16.51%) |  |
| Grade IV | 6(3.08%) | 1534(2.65%) |  | 6(3.59%) | 12(3.74%) |  |
| Unknown | 73(37.43%) | 6357(10.99%) |  | 59(35.33%) | 117(36.45%) |  |
| Histology |  |  | <0.001 |  |  | 0.178 |
| AC | 172(88.21%) | 51579(89.18%) |  | 146(87.42%) | 274(85.36%) |  |
| MC | 4(2.05%) | 3833(6.63%) |  | 4(2.40%) | 22(6.85%) |  |
| SRCC | 4(2.05%) | 615(1.07%) |  | 3(1.80%) | 5(1.56%) |  |
| Other | 15(7.69%) | 1807(3.12%) |  | 14(8.38%) | 20(6.23%) |  |
| pT |  |  | <0.001 |  |  | 0.061 |
| T1-2 | 34(17.44%) | 17519(30.29%) |  | 31(18.56%) | 38(11.84%) |  |
| T3-4 | 76(38.97%) | 36520(63.15%) |  | 71(42.52%) | 166(51.71%) |  |
| Unknown | 85(43.59%) | 3795(6.56%) |  | 65(38.92%) | 117(36.45%) |  |
| pN |  |  | <0.001 |  |  | 0.457 |
| N0-N1b | 95(48.72%) | 46957(81.19%) |  | 84(50.30%) | 155(48.29%) |  |
| N2a-2b | 28(14.36%) | 8014(13.86%) |  | 28(16.77%) | 44(13.71%) |  |
| Unknown | 72(36.92%) | 2863(4.95%) |  | 55(32.93%) | 122(38.00%) |  |
| Bone metastases |  |  | <0.001 |  |  | 0.170 |
| No/Unknown | 154(78.97%) | 57302(99.08%) |  | 138(82.63%) | 280(87.23%) |  |
| Yes | 41(21.03%) | 532(0.92%) |  | 29(17.37%) | 41(12.77%) |  |
| Liver metastases |  |  | <0.001 |  |  | 0.767 |
| No/Unknown | 76(38.97%) | 49905(86.29%) |  | 71(42.51%) | 132(41.12%) |  |
| Yes | 119(61.03%) | 7929(13.71%) |  | 96(57.49%) | 189(58.88%) |  |
| Lung metastases |  |  | <0.001 |  |  | 0.744 |
| No/Unknown | 94(48.21%) | 55196(95.44%) |  | 90(53.89%) | 168(52.34%) |  |
| Yes | 101(51.79%) | 2638(4.56%) |  | 77(46.11%) | 153(47.66%) |  |
| CEA |  |  | <0.001 |  |  | 0.125 |
| Negative/Unknown | 78(40.00%) | 41425(71.63%) |  | 69(41.32%) | 110(34.27%) |  |
| Positive | 117(60.00%) | 16409(28.37%) |  | 98(58.68%) | 211(65.73%) |  |
| Perineural Invasion |  |  | 0.174 |  |  | 0.903 |
| None/Unknown | 182(93.33%) | 52322(90.47%) |  | 154(92.22%) | 295(91.90%) |  |
| Present | 13(6.67%) | 5512(9.53%) |  | 13(7.78%) | 26(8.10%) |  |
| Surgery |  |  | <0.001 |  |  | 0.587 |
| None/unknown | 139(71.28%) | 7648(13.22%) |  | 112(67.07%) | 223(69.47%) |  |
| Performed | 56(28.72%) | 50186(86.78%) |  | 55(32.93%) | 98(30.53%) |  |
| Radiotherapy |  |  | <0.001 |  |  | 0.385 |
| None/unknown | 66(33.85%) | 50133(86.68%) |  | 66(39.52%) | 140(43.61%) |  |
| Performed | 129(66.15%) | 7701(13.32%) |  | 101(60.48%) | 181(56.39%) |  |
| Systematic therapy |  |  | 0.096 |  |  | 0.448 |
| None | 127(65.13%) | 36560(63.22%) |  | 116(69.46%) | 235(73.21%) |  |
| Before surgery | 7(3.60%) | 4449(7.69%) |  | 7(4.19%) | 17(5.30%) |  |
| After surgery | 61(31.27%) | 16825(29.09%) |  | 44(26.35%) | 69(21.49%) |  |

Abbreviations: AC adenocarcinoma, MC Mucinous adenocarcinoma, and SRCC Signet ring cell carcinoma.

^∗^ P values were made by Chi square-test.

^†^ Other=American Indian/AK Native, and Asian/Pacific Islander
