# Supplementary material for: Risk Factors on the Incidence and Prognostic Effects of Colorectal Cancer With Brain Metastasis: A SEER-Based Study
Source: Front Oncol. 2022 Mar 18;12:758681. doi: 10.3389/fonc.2022.758681 (PMC8971714; doi:10.3389/fonc.2022.758681)
Supplement: Supplementary Table 3 — Univariable analyses using Cox models associated with CSS and OS for patients with CRC after PSM. [file Table_3.docx]

**Supplementary Table 3** Univariable analyses using Cox models associated with CSS and OS for patients with CRC after PSM.

|  | CSS | | | OS | | |
| --- | --- | --- | --- | --- | --- | --- |
|  | HR | 95%CI | P | HR | 95%CI | P |
| Brain metastasis |  |  | <0.001 |  |  | <0.001 |
| None | 1 |  |  | 1 |  |  |
| Yes | 2.017 | 1.626-2.503 |  | 1.923 | 1.560-2.371 |  |
| Age(years) |  |  | <0.001 |  |  | <0.001 |
| <50 | 1 |  |  | 1 |  |  |
| 50-59 | 1.147 | 0.824-1.596 |  | 1.155 | 0.837-1.596 |  |
| 60-69 | 1.452 | 1.056-1.996 |  | 1.439 | 1.054-1.965 |  |
| 70-79 | 1.585 | 1.108-2.266 |  | 1.710 | 1.214-2.410 |  |
| ≥80 | 2.444 | 1.700-3.514 |  | 2.698 | 1.908-3.817 |  |
| Race |  |  | 0.446 |  |  | 0.213 |
| White | 1 |  |  | 1 |  |  |
| Black | 1.168 | 0.875-1.560 |  | 1.271 | 0.970-1.666 |  |
| Other† | 1.166 | 0.817-1.666 |  | 1.096 | 0.768-1.563 |  |
| Gender |  |  | 0.358 |  |  | 0.178 |
| Male | 1 |  |  | 1 |  |  |
| Female | 0.909 | 0.741-1.114 |  | 0.874 | 0.718-1.064 |  |
| Location |  |  | 0.004 |  |  | 0.003 |
| Right side | 1 |  |  | 1 |  |  |
| Left side | 0.640 | 0.478-0.856 |  | 0.668 | 0.507-0.880 |  |
| Rectum | 0.761 | 0.607-0.954 |  | 0.726 | 0.583-0.905 |  |
| Grade |  |  | 0.074 |  |  | 0.035 |
| Grade I | 1 |  |  | 1 |  |  |
| Grade II | 1.241 | 0.581-2.649 |  | 1.173 | 0.577-2.387 |  |
| Grade III | 1.433 | 0.658-3.122 |  | 1.292 | 0.622-2.687 |  |
| Grade IV | 0.675 | 0.256-1.780 |  | 0.591 | 0.232-1.503 |  |
| Unknown | 1.523 | 0.712-3.257 |  | 1.471 | 0.722-2.998 |  |
| Histology |  |  | 0.247 |  |  | 0.518 |
| AC | 1 |  |  | 1 |  |  |
| MC | 0.722 | 0.437-1.193 |  | 0.767 | 0.477-1.232 |  |
| SRCC | 0.737 | 0.323-1.680 |  | 0.687 | 0.301-1.565 |  |
| Other | 0.700 | 0.440-1.111 |  | 0.874 | 0.582-1.312 |  |
| pT |  |  | <0.001 |  |  | <0.001 |
| T1-2 | 1 |  |  | 1 |  |  |
| T3-4 | 0.681 | 0.499-0.929 |  | 0.706 | 0.522-0.955 |  |
| Unknown | 1.377 | 1.007-1.883 |  | 1.381 | 1.019-1.872 |  |
| pN |  |  | 0.005 |  |  | 0.006 |
| N0-N1b | 1 |  |  | 1 |  |  |
| N2a-N2b | 0.932 | 0.679-1.279 |  | 0.965 | 0.714-1.302 |  |
| Unknown | 1.389 | 1.115-1.731 |  | 1.376 | 1.112-1.703 |  |
| Bone metastasis |  |  | <0.001 |  |  | <0.001 |
| No/Unknown | 1 |  |  | 1 |  |  |
| Yes | 1.863 | 1.420-2.444 |  | 1.806 | 1.386-2.353 |  |
| Liver metastasis |  |  | <0.001 |  |  | <0.001 |
| No/Unknown | 1 |  |  | 1 |  |  |
| Yes | 2.226 | 1.781-2.783 |  | 2.047 | 1.654-2.533 |  |
| Lung metastasis |  |  | <0.001 |  |  | <0.001 |
| No/Unknown | 1 |  |  | 1 |  |  |
| Yes | 1.600 | 1.302-1.967 |  | 1.486 | 1.218-1.814 |  |
| CEA |  |  | 0.001 |  |  | 0.028 |
| Negative/Unknown | 1 |  |  | 1 |  |  |
| Positive | 1.329 | 1.068-1.653 |  | 1.263 | 1.025-1.556 |  |
| Perineural Invasion |  |  | 0.027 |  |  | 0.020 |
| Not present/Unknown | 1 |  |  | 1 |  |  |
| Present | 0.641 | 0.432-0.950 |  | 0.637 | 0.436-0.931 |  |
| Surgery |  |  | <0.001 |  |  | <0.001 |
| None/unknown | 1 |  |  | 1 |  |  |
| Performed | 0.422 | 0.331-0.538 |  | 0.430 | 0.340-0.543 |  |
| Radiotherapy |  |  | 0.081 |  |  | 0.023 |
| None/unknown | 1 |  |  | 1 |  |  |
| Performed | 0.833 | 0.678-1.023 |  | 0.795 | 0.652-0.969 |  |
| Systematic therapy |  |  | <0.001 |  |  | <0.001 |
| None | 1 |  |  | 1 |  |  |
| Before surgery | 0.301 | 0.172-0.527 |  | 0.278 | 0.159-0.485 |  |
| After surgery | 0.460 | 0.353-0.598 |  | 0.450 | 0.349-0.581 |  |

Abbreviations: AC adenocarcinoma, MC Mucinous adenocarcinoma, and SRCC Signet ring cell carcinoma.

† Other=American Indian/AK Native, and Asian/Pacific Islander
