# Supplementary material for: Risk Factors on the Incidence and Prognostic Effects of Colorectal Cancer With Brain Metastasis: A SEER-Based Study
Source: Front Oncol. 2022 Mar 18;12:758681. doi: 10.3389/fonc.2022.758681 (PMC8971714; doi:10.3389/fonc.2022.758681)
Supplement: Supplementary Table 4 — Univariable Cox analysis of prognostic factors associated with CSS and OS for CRCBM patients after PSM. [file Table_4.docx]

**Supplementary Table 4** Univariable analyses using Cox models associated with CSS and OS for patients with CRC after PSM.

|  | CSS | | | OS | | |
| --- | --- | --- | --- | --- | --- | --- |
|  | HR | 95%CI | P^*^ | HR | 95%CI | P^∗^ |
| Age(years) |  |  | <0.001 |  |  | <0.001 |
| <50 | 1 |  |  | 1 |  |  |
| 50-59 | 1.302 | 0.734-2.310 |  | 1.272 | 0.726-2.228 |  |
| 60-69 | 1.365 | 0.773-2.410 |  | 1.374 | 0.790-2.389 |  |
| 70-79 | 2.138 | 1.154-3.961 |  | 2.036 | 1.110-3.736 |  |
| ≥80 | 4.226 | 2.207-8.090 |  | 4.399 | 2.346-8.250 |  |
| Race |  |  | 0.541 |  |  | 0.673 |
| White | 1 |  |  | 1 |  |  |
| Black | 0.923 | 0.545-1.565 |  | 1.998 | 0.605-1.645 |  |
| Other^†^ | 1.353 | 0.759-2.411 |  | 1.296 | 0.728-2.307 |  |
| Gender |  |  | 0.046 |  |  | 0.039 |
| Male | 1 |  |  | 1 |  |  |
| Female | 0.709 | 0.505-1.995 |  | 0.705 | 0.506-0.982 |  |
| Location |  |  | 0.260 |  |  | 0.369 |
| Right side | 1 |  |  | 1 |  |  |
| Left side | 0.684 | 0.434-1.079 |  | 0.736 | 0.475-1.139 |  |
| Rectum | 0.864 | 0.591-1.262 |  | 0.856 | 0.589-1.244 |  |
| Grade |  |  | 0.622 |  |  | 0.564 |
| Grade I | 1 |  |  | 1 |  |  |
| Grade II | 0,816 | 0.112-5.916 |  | 0.841 | 0.116-6.095 |  |
| Grade III | 0.952 | 0.129-7.013 |  | 0.985 | 0.134-7.254 |  |
| Grade IV | 1.448 | 0.174-12.075 |  | 1.452 | 0.174-12.105 |  |
| Unknown | 1.040 | 0.143-7.587 |  | 1.113 | 0.153-8.101 |  |
| Histology |  |  | 0.264 |  |  | 0.352 |
| AC | 1 |  |  | 1 |  |  |
| MC | 0.939 | 0.295-2.991 |  | 0.891 | 0.280-2.834 |  |
| SRCC | 2.749 | 1.006-7.511 |  | 2.816 | 0.888-8.929 |  |
| Other | 0.947 | 0.480-1.869 |  | 1.145 | 0.616-2.129 |  |
| pT |  |  | 0.032 |  |  | 0.033 |
| T1-2 | 1 |  |  | 1 |  |  |
| T3-4 | 1.113 | 0.686-1.808 |  | 1.117 | 0.695-1.795 |  |
| Unknown | 1.707 | 1.044-2.792 |  | 1.691 | 1.044-2.738 |  |
| pN |  |  | 0.330 |  |  | 0.293 |
| N0-N1b | 1 |  |  | 1 |  |  |
| N2a-N2b | 1.022 | 0.649-1.607 |  | 1.065 | 0.686-1.654 |  |
| Unknown | 1.326 | 0.902-1.951 |  | 1.348 | 0.923-1.968 |  |
| Bone metastasis |  |  | 0.028 |  |  | 0.029 |
| No/Unknown | 1 |  |  | 1 |  |  |
| Yes | 1.611 | 1.052-2.465 |  | 1.594 | 1.050-2.420 |  |
| Liver metastasis |  |  | 0.020 |  |  | 0.024 |
| No/Unknown | 1 |  |  | 1 |  |  |
| Yes | 1.514 | 1.067-2.147 |  | 1.481 | 1.053-2.082 |  |
| Lung metastasis |  |  | 0.083 |  |  | 0.104 |
| No/Unknown | 1 |  |  | 1 |  |  |
| Yes | 1.348 | 0.961-1.890 |  | 1.316 | 0.945-1.832 |  |
| CEA |  |  | 0.878 |  |  | 0.970 |
| Negative/Unknown | 1 |  |  | 1 |  |  |
| Positive | 1.027 | 0.729-1.449 |  | 1.006 | 0.719-1.408 |  |
| Perineural Invasion |  |  | 0.469 |  |  | 0.549 |
| Not present/Unknown | 1 |  |  | 1 |  |  |
| Present | 0.787 | 0.413-1.503 |  | 0.828 | 0.446-1.535 |  |
| Surgery |  |  | <0.001 |  |  | <0.001 |
| None/unknown | 1 |  |  | 1 |  |  |
| Performed | 0.510 | 0.351-0.740 |  | 0.512 | 0.356-0.737 |  |
| Radiotherapy |  |  | 0.139 |  |  | 0.109 |
| None/unknown | 1 |  |  | 1 |  |  |
| Performed | 0.773 | 0.549-1.088 |  | 0.761 | 0.545-1.063 |  |
| Systematic therapy |  |  | <0.001 |  |  | <0.001 |
| None | 1 |  |  | 1 |  |  |
| Before surgery | 0.154 | 0.048-0.492 |  | 0.150 | 0.047-0.481 |  |
| After surgery | 0.402 | 0.268-0.604 |  | 0.426 | 0.288-0.630 |  |

Abbreviations: AC adenocarcinoma, MC Mucinous adenocarcinoma, and SRCC Signet ring cell carcinoma.

^∗^ P values were calculated by multivariate logistic regression after adjusted by age, gender, race, location, grade, histology, pT, pN, bone metastases, liver metastases, lung metastases, CEA and perineural invasion.

^†^ Other=American Indian/AK Native, and Asian/Pacific Islander
